# Robust extraction of quantitative structural information from high-variance histological images of livers from necropsied Soay sheep

**DOI:** 10.1098/rsos.170111

**Published:** 2017-07-19

**Authors:** Q. Caudron, R. Garnier, J. G. Pilkington, K. A. Watt, C. Hansen, B. T. Grenfell, T. Aboellail, A. L. Graham

**Affiliations:** 1 Department of Ecology and Evolutionary Biology, Princeton University, Princeton, NJ, USA; 2 Institute of Evolutionary Biology, School of Biological Sciences, University of Edinburgh, Edinburgh, UK; 3 Department of Microbiology, Immunology and Pathology, Colorado State University, Fort Collins, CO, USA

**Keywords:** disease ecology, quantitative histology, *Ovis aries*, computer-aided diagnostics, histopathology

## Abstract

Quantitative information is essential to the empirical analysis of biological systems. In many such systems, spatial relations between anatomical structures is of interest, making imaging a valuable data acquisition tool. However, image data can be difficult to analyse quantitatively. Many image processing algorithms are highly sensitive to variations in the image, limiting their current application to fields where sample and image quality may be very high. Here, we develop robust image processing algorithms for extracting structural information from a dataset of high-variance histological images of inflamed liver tissue obtained during necropsies of wild Soay sheep. We demonstrate that features of the data can be measured in a fully automated manner, providing quantitative information which can be readily used in statistical analysis. We show that these methods provide measures that correlate well with a manual, expert operator-led analysis of the same images, that they provide advantages in terms of sampling a wider range of information and that information can be extracted far more quickly than in manual analysis.

## 1. Introduction

Imaging is nowadays a valued method for data acquisition in biology [1]. Able to capture form, structure, pattern and texture, imaging data present a potentially rich repertoire of information not always available to other sampling modalities. The use of imaging is well established in human and veterinary clinical research and hospital practice settings, but the required equipment still limits the use of imaging in field biology and ecology. However, the growing availability of inexpensive, high-quality sensors makes images an attractive source of data in many experimental and clinical settings.

Prior to the considerable advances in computer vision over the last decade, much analysis of image data required operator input, with most measurements typically taking the form of qualitative descriptions or semi-quantitative scores. Such scoring systems are widely used to extract diagnostic and prognostic information in histopathology, where this type of information has a clear clinical interpretation. However, these scoring methods are known to contain inconsistencies owing partly to operator interpretation bias [2,3]. In addition, the data do not lend themselves well to rigorous statistical analysis, due to their discrete or ordinal nature. Finally, manual scoring typically requires extensive training, and is costly and time-consuming. Similar difficulties in the extraction of quantitative data from images are prevalent across a number of fields of biology, including ecology [4–7].

Much of the recent progress in image processing methodology has been driven by an increased demand for automated, robust image analysis methods in the past decade [1,8]. These systems aim to rapidly provide reproducible, quantitative and standardized information, extracted over the entire image, for analysis or interpretation. Image processing algorithms are also able to quantify information that may be difficult or impossible for the human eye to measure with accuracy, such as tissue porosity [9], texture [10] or subtle differences in shape or structure [11,12]. In clinical settings, image analysis algorithms have contributed to both speed and accuracy of the diagnostic process, challenging the traditional paradigm of manual inspection of radiographs or slides [13–15]. Automated image processing methods have also been successfully developed in research, where they span an incredibly diverse set of applications, including cell tracking, morphometric analysis and automated species recognition; for a non-exhaustive review, see Rittscher [16].

A major challenge in the development of automated image processing algorithms is that of variability in the process leading up to image capture [17]. Discrepancies in sample preservation and preparation or during image capture can cause significant difficulties in parameterizing algorithms for the consistent extraction of information. Therefore, care must be taken to ensure that such variations do not affect what is being measured and to avoid artefacts commonly found in tissue sections which are improperly preserved or processed. As such, much of the image processing work done on microscopy to date has been applied in laboratory settings, where this variance can be limited by careful, or automated, processing. However, not all settings offer such strict control over sample quality or preparation. In field biology, for instance, tissue samples may deteriorate due to harsh environmental conditions, or due to field constraints delaying the access to a laboratory in which samples can be processed for histological analysis. Histopathological image datasets originating from ecological studies may thus present considerable variation in luminosity, colour and contrast, not just between but also within individual images [18].

Despite the inconsistencies, these datasets are likely to contain valuable information about the underlying biological processes (such as the origin of disease or the cause of death) and may greatly contribute to experimental and investigative work [19]. In this article, we demonstrate that quantitative, structural information can be robustly extracted from a high-variance dataset of histological slides. We begin by describing the preparation and imaging of liver tissue originating from a wild population of Soay sheep. Then, we define the structural information to be extracted and the algorithms used to measure them. We present the validation of our algorithms against a dataset of measures generated by a manual operator. Finally, we discuss the potential for automated image processing methods in systems where the generated data may contain significant variation.

## 2. Data

### 2.1. Field sampling

We obtained samples from a wild Soay sheep population located on the island of Hirta, St. Kilda (Scotland) which has been the subject of a long-term ecological study. The population was established from nearby Soay island in the 1930s and has been completely unmanaged since then. Since 1985, individual sheep have been tagged so that survival, reproduction and various aspects of phenotype can be monitored longitudinally [20]. The population exhibits unstable dynamics with occasional population crashes in which up to 60% of the sheep die of some combination of malnutrition, infections and exposure to inclement weather conditions in late winter [21]. Liver lesions consistent with these different and occasionally overlapping causes of death have been reported following gross pathological evaluation performed on the field [22]. Throughout the time of peak mortality during the crash of winter 2011–2012, the study area was checked daily for dead tagged individuals. Sheep were thus found within 24 h of death and immediately necropsied. Liver samples (2 cm^3^) were consistently collected from the edge of the upper right hepatic lobe and fixed in 5% neutral buffered formalin. A total of 143 Soay sheep were sampled, a subset of which is used in the present paper (see below).

### 2.2. Laboratory procedure

Owing to seasonally restricted boat access and weight-restricted helicopter transport to and from Hirta, the samples could only be brought off the island for histological processing the following August. Because of this delay, tissue samples showed signs of overfixation when processed [23]. Briefly, samples were first processed overnight in an automatic tissue processor for paraffin inclusion (Leica ASP 300S, Leica, Germany). Samples were then embedded in paraffin (Tissue-Tek embedding center, Miles Scientific, Newark, DE, USA) and sectioned using a rotary microtome (Leica RM2255, Leica, Germany). Because of the fragility of the samples, sectioning was performed at 10 µm. The resulting sections were mounted on transparent glass slides, dried overnight and stained with a routine haematoxylin and eosin protocol (Hematoxylin and Eosin Staining kit, Scytek Laboratories, Logan, UT, USA). After drying, slides were mounted (Organo/Limonene Mount, Sigma-Aldrich, St. Louis, MO, USA) and sealed with clear nail polish.

Image capture was performed in RAW format using a Canon 600D digital SLR, on a Nikon Eclipse 80i brightfield microscope. An adapter requiring the removal of both the lens of the camera and the ocular of the microscope was used, and only the objective lenses (4× and 40×) of the microscope were used. The camera was connected to a laptop via USB to allow triggering the camera without introducing vibrations. Finally, the camera was set on the aperture-priority mode, with ISO set to 100, and the focus was made using the microscope, sequentially at different levels of zoom of the camera. Images have a resolution of 3456 by 5184 pixels, with a depth of 14 bits per channel. This bit depth allows for a much more continuous luminosity curve (with 16 834 levels) than the traditional, 8 bits set-up (corresponding to 256 luminosity values). Areas of similar luminosity or colour can thus be better differentiated, for more accurate and robust segmentation and region detection. Similarly, the large spatial resolution of the image offers more capacity to accurately resolve structures in the image while ensuring a large frame of view. Finally, the high resolution and large bit depth provide a large scope for preprocessing without introducing noise or creating artefacts. To ensure a uniform spectral coverage, the microscope's light was set to maximum intensity. During image capture, care was taken to avoid any area of the slide that would show signs of artefactual damage, for instance due to overfixation.

To quantify aspects of infection-, inflammation- and malnutrition-associated liver disease processes in the Soay sheep, we focused our analysis on a small number of features likely to provide insights on immune [24,25] and nutritional [22] markers related to survival in this population. We primarily aimed to characterize and quantify inflammatory processes and hepatic cell densities. The overall size of inflammatory foci and the number of constituent inflammatory cells serve as indicators of the degree, distribution and duration of hepatic lesions. We thus used these features as proxies for the focal or portal inflammation scores in the Ishak grading scale [26], commonly used to assess the extent of liver pathologies. We also calculated the density of cells outside of inflammatory zones, to determine whether the parenchyma of the liver was enlarged due to acute cell swelling or had shrunk due to nutritional atrophy.

**Figure 1.**
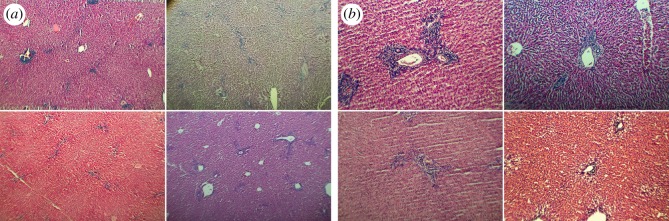
High-variance liver histological images from necropsied Soay sheep. Examples of images in the dataset, demonstrating high variability in staining, contrast, luminosity and tissue quality. (*a*) Widefield images and (*b*) narrowfield images.

To effectively capture and validate our measures against an operator-led analysis, we used two levels of magnification to capture these lesions. At the widefield level (2 × 3 mm per image), we captured fields of view containing continuous tissue with a number of inflammatory foci and minimal obvious artefactual distortions (such as tears in the tissue). The goal is to analyse the spatial extent of inflammatory foci. Specifically, every pixel in an image was assigned a binary value by our algorithm identifying it as either part of an inflammatory zone or not, yielding the spatial distribution of inflammatory zones, as well as their sizes. Narrowfield images (0.8 mm × 1.2 mm per view) were captured centred on a single inflammatory focus (not necessarily included in a widefield image), to enable finer measurement of focus surface area, as well as of the spatial distribution of nuclei in the background tissue, outside of the inflammatory focus. No foci in the narrowfield images were so large that they could not be fully captured. Figure 1 shows example images at both magnifications. At the widefield level, each pixel represents an area of 0.33 µm^2^. Pixels at the narrowfield level had a surface area of 0.053 µm^2^.

General preprocessing was applied to all images upon capture. Global preprocessing involved increasing contrast via a sigmoid transform, increasing colour saturation, noise reduction and sharpening using an unsharp mask [27]. More specific preprocessing was targeted at making the structural information of interest (for example, the inflammatory foci) more obvious in the images. As such, the intensity of yellow and orange hues was increased, while their saturation was decreased to improve separation of the tissue from the background, and blues and purples were strongly saturated and darkened to emphasize nuclei and inflammatory foci. All preprocessing is entirely non-destructive: as images are stored in RAW format with a separate file containing preprocessing metadata, the original, straight-out-of-camera images are kept separate from their preprocessing, which could therefore be fine-tuned at any stage for optimal results. Images were then exported as high-quality 8-bit JPEG images for processing.

A manual, operator-led analysis was performed, providing the ground truth against which the results of the automatic image processing algorithms were compared. To obtain this, individual, preprocessed images were presented to a trained operator, first at the widefield level, where the operator used point and click methods to define the contours of all inflammatory zones in each image. Then, narrowfield images were presented one by one, and the contour of the central inflammatory focus was defined, as in the widefield images (albeit with finer spatial resolution). The operator further selected a rectangular area outside the focus deemed representative of the background tissue in order to measure adaptive changes in hepatic parenchyma. Within that rectangle, the operator then clicked on each nucleus individually. In this manner, the locations and surface areas of inflammatory foci were captured at the widefield level, as well as the finer boundary of a single inflammatory zone in the narrowfield images. Additionally, the density and spatial distribution of cells were captured in narrowfield images.

For the present paper, the manual operator was presented with images from 100 randomly selected sheep at both the widefield and at the narrowfield views. In total, 81 images at the widefield and 88 images at the narrowfield were analysed: the manual operator could not select representative areas in the remaining images. This is because, is some cases, haematoxylin staining was weak and impaired the detection by the human operator of the portal spaces at the widefield and of individual cells' nuclei at the narrowfield. In other cases, the images contained large tears, due in large part to unavoidable overfixation due to the nature of the sampling, that made it difficult for the operator to select inflammatory foci. However, the pre- and post-processing algorithms were sufficiently robust to allow the automatic processing of such images. Because we are interested in associations between the results of the manual operator and the algorithm, we do not report the algorithm results for these 19 widefield and 12 narrowfield images here.

## 3. Image processing

Images at the widefield were next processed to separate the inflammatory foci and the veins in the images from the rest of the tissue, a process known as segmentation [27]. Parameters for these image processing algorithms can be found in the electronic supplementary material. Owing to computational costs, widefield algorithms are applied on images scaled down to a quarter of their original resolution.

**Figure 2.**
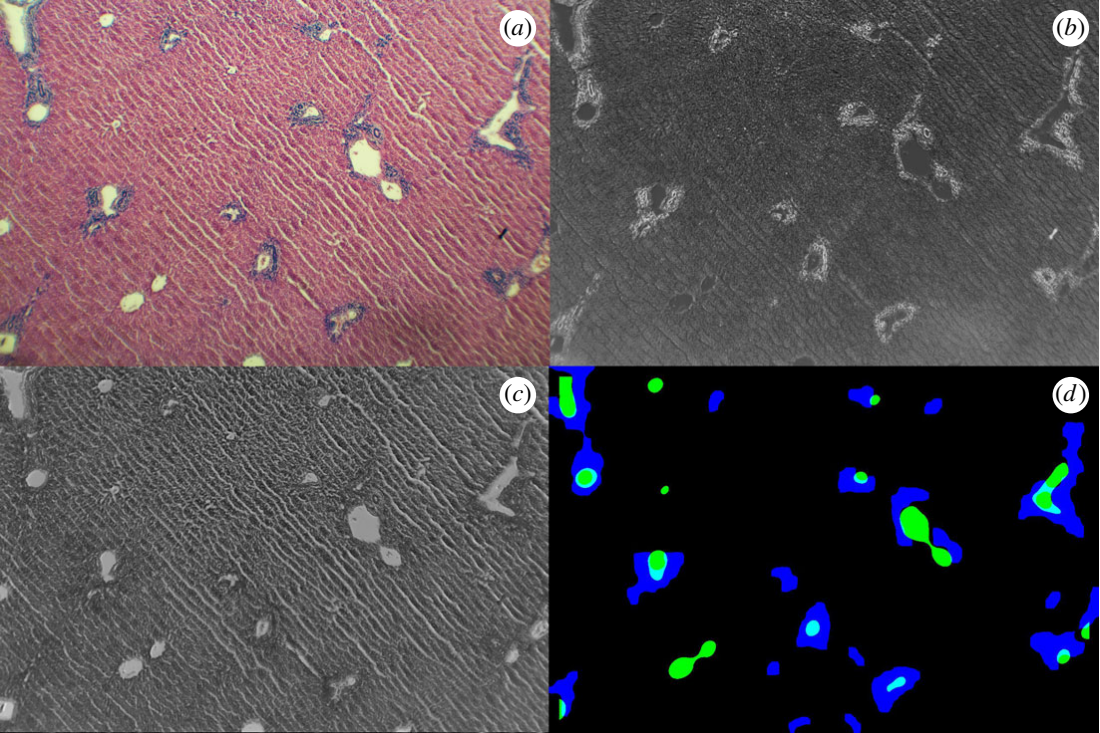
Use of colour deconvolutions to segment veins and inflammatory foci at the widefield. (*a*) Original image. (*b*) After colour deconvolution, the haematoxylin channel highlights areas of high nuclear concentration. (*c*) Dividing the unstained colour channel by the eosin channel results in bright areas highlighting veins and other areas with no tissue. (*d*) After segmentation, regions of inflammation are identified in blue, and veins are identified in green.

Figure 2 shows an example of the steps of the widefield processing algorithm. From the preprocessed image (figure 2*a*), we first performed a colour deconvolution [28]. This process attempts to quantify how much a particular pixel has been stained by haematoxylin or by eosin by transforming the image into an orthogonal representation of stain densities. Parameters for colour deconvolution were obtained by imaging slides stained only by haematoxylin or only by eosin, from which a deconvolution filter can be constructed. After deconvolution, three colour channels remain, analogous to the Red–Green–Blue (RGB) channels of standard colour images; however, these channels represent Haematoxylin-Eosin-Unstained (HEU) instead. For instance, a pixel with a large value in the Haematoxylin channel is a pixel that has absorbed a large quantity of haematoxylin, and is therefore probably part of a nucleus.

From the HEU image, we can identify regions of inflammation by looking at areas with high haematoxylin content, as this dye primarily stains DNA; areas rich in haematoxylin are therefore regions of the image that contain high nuclear density (and thus a higher density of cells altogether). Because immune cells have a higher nucleus to cytoplasm ratio, these regions are indicative of a high density of immune cells. Starting with the H channel (figure 2*b*), we applied contrast-limited adaptive histogram equalization [29] to the greyscale image to improve contrast and to remove any global gradients, such as those that may be caused by non-uniform lighting from the microscope's light source. We also applied a Gaussian blur with a standard deviation of 5 pixels to remove noise and generate a smooth surface, to allow for smooth contours to be found in thresholding. After applying a sigmoid transform to increase contrast [27], we applied an adaptive thresholding operation, resulting in a black and white image, highlighting areas that are strongly stained with haematoxylin. We then removed small, connected components of less than 250 contiguous pixels, discarding them as noise. Finally, a maximum filter of size 11 pixels was applied, followed by a morphological closing with a disc of size 15 pixels. The result can be directly compared to the operator measures, validating whether areas found to be inflammatory by the algorithm match those found manually.

Similarly, we can segment veins from the image by localizing areas that contain little dye and instead have high values in the Unstained channel. We take the ratio between the U and the E channels to characterize how unstained a pixel is compared to the background tissue. The result (figure 2*c*) has bright areas where the pixels are mostly unstained and contain little eosin, and are characteristic of veins or gaps in the tissue. From this starting point, and in the same manner as in the identification of inflammatory zones, we apply a slight Gaussian blur with a standard deviation of 11 pixels to remove noise and provide smooth contours, followed by an adaptive thresholding to segment out the veins in the image. We finish with a morphological closing with a disc of size 25 pixels and by removing small connected components of 200 pixels or smaller.

Figure 2*d* shows the results of these segmentation procedures, with inflammatory zones shown in blue and veins shown in green. Comparing with the original image in figure 2*a*, we identify a number of inflammatory foci and veins, and note that small tears in the tissue were not identified as veins due to careful calibration of the steps in the algorithm.

**Figure 3.**
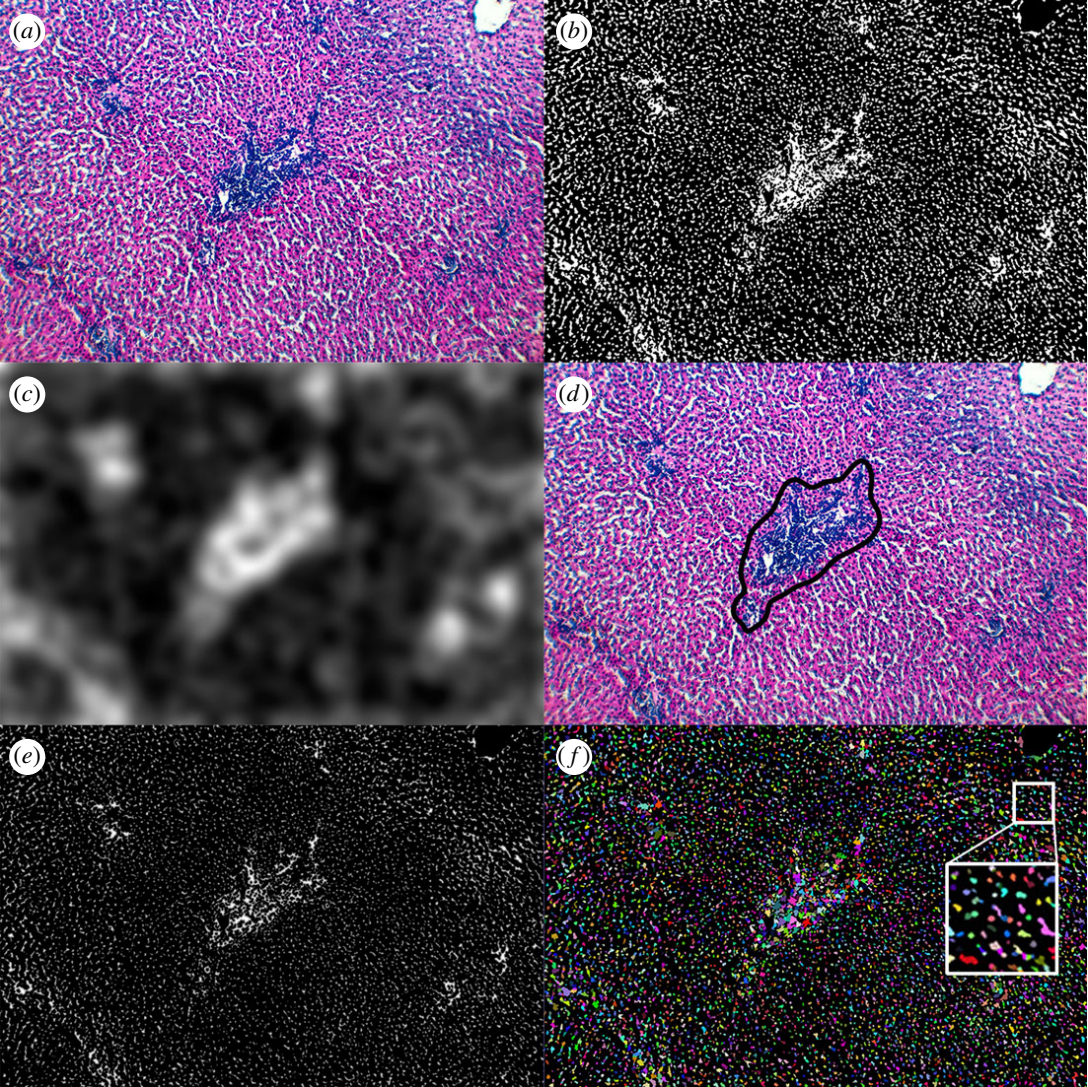
Image processing steps to identify inflammatory foci and segment nuclei at the narrowfield. (*a*) Original image. (*b*) After a sigmoid transform for contrast, an adaptive thresholding, a binary closing and removing noise, nuclei are in white and everything else is black. (*c*) Combining local density with entropy yields an image where bright areas indicate high concentrations of nuclei. (*d*) The contour of the inflammatory focus in the image is detected. (*e*) The brightest points are those furthest away from the background as a result of a Euclidean distance transform on (*b*). (*f*) The image is fully segmented, with separate nuclei indicated by different colours (*f*).

The progression of the image processing algorithms for narrowfield images is exemplified in figure 3. From the preprocessed image (figure 3*a*), we began by discarding the blue and green channels. Nuclei have very dark luminosity in the red channel from the haematoxylin, while empty space and cells have high luminosity in that same channel. Dropping all but the red channel results in a large dimensionality reduction (and thus algorithm speed gain) while still ensuring accurate segmentation. This resulted in a greyscale image where, due to their very dark blue colour, nuclei appear very dark. By contrast, the pixels making up the cytoplasm or sinusoidal blood will appear light, due to their red content. Other pixels in the red channel are typically very light; these make up the cytoplasm, veins/vessels, or sinusoids, all of which contain high intensities of red in the original image. We applied a sigmoid transform to further boost contrast, and then took an adaptive threshold to binarize the image, leaving black pixels representing nuclei and white for any other tissue or background pixels. To eliminate small holes in the white regions while ensuring smooth surfaces for the watershed algorithm, we reconnected small components using a morphological closing operation using a disc of radius 3 as the structuring element [27], enlarging the boundaries of regions of white pixels before shrinking them again. We chose a disc as the structuring element to resemble the target structure shape, and avoid artificially pixelating the newly created image (which would have happened with other structuring elements such as diamonds or squares). The morphological closing greatly reduced small elements of noise and aided in smoothing the contours of nuclei. We finished by removing any remaining small, connected components of 100 contiguous pixels or less, and inverting the image such that nuclei are white and other tissue or background pixels are black. Figure 3*b* shows an example image at this stage of processing.

From here, we applied two separate sets of operations to this clean, binary image: the first extracted the contour of the central inflammatory focus, and the second segmented individual nuclei such that they can be counted and localized. For the central focus, we computed the local entropy [30], a measure of the quantity of information, or randomness, in an image. Local entropy was nonlinearly combined with a Gaussian-blurred version of our clean image (see the electronic supplementary material for details). The Gaussian filtered image is a strongly blurred image obtained with a 61 pixel standard deviation, and provides a measure of cellular density. Entropy is calculated in a 3 pixel radius disc as a base-2 logarithm, and then blurred using a Gaussian kernel with a standard deviation of 75 pixels. The combination of these two quantities (figure 3*c*) provided a measure of local structure that also coincided with high densities of nuclei. These areas are likely to be inflammatory zones—due to their more irregular packing and greater cellular overlap, these regions contain more local structures such as bile ducts. Following processing, the resulting images had a generally bimodal histogram of pixel intensities. We applied an Otsu threshold [30,31] to separate areas likely to be inflammatory foci from those that are not. Using a marching squares algorithm [27,32], we found the contours of the most central and largest object in the image. An example of a detected contour can be found in figure 3*d*.

Because of the 10 µm thickness of our slices in the *z*-dimension, nuclei were likely to overlap, especially inside the inflammatory focus. We attempted to separate them using a watershed algorithm [30,33]. We began by taking a Euclidean distance transform [34] of our clean, binary image. This replaces all white pixels with a value between 0 and 1 according to their distance from the nearest black pixel. Thus, white pixels that are on the edge of a region receive a value of zero, while pixels that are in the middle of nuclei will receive large values. This is analogous to seeing the image as a landscape, where black pixels form the ground at sea level, and individual nuclei each are peaks, or mountains. If two nuclei overlap, but not so much that the centre of one is inside the boundary of another, they will form twin peaks. The Euclidean transform of the clean, binary image in figure 3*b* can be seen in figure 3*e*. Using the distance-transformed image, we extracted the location of all peaks across the image and used these to seed a watershed algorithm to segment overlapping nuclei. The result is shown in figure 3*f*, where each nucleus is given a random colour to demonstrate that connected regions of overlapping nuclei can be segmented.

## 4. Validation measures

To validate the information extracted by our image processing algorithms, we calculated a number of scores to be compared with the operator-scored images. As such,

— let *I* denote the binary image, generated by our algorithm, where white pixels represent zones of inflammation, and black pixels represent background tissue (see electronic supplementary material, figure S1 for an example),
— let *T* denote the binary image generated by the human operator, with zones of inflammation and background denoted as in *I* (see electronic supplementary material, figure S1 for an example)*,*
— denote individual pixels in an image according to their subscripts, such that *A_ij_* is the pixel in the *i*th row and *j*th column of *A*,
— let *S* denote the number of pixels in an image, such that *S *= *i*_max_ × *j*_max_ for an image with *i*_max_ rows and *j*_max_ columns,
— let ⊗ denote the logical conjunction operator between images, such that *A* ⊗ *B* is a binary image with the same dimensions as *A* and *B*, where a pixel is white if that pixel was white in both *A* and in *B*, and black otherwise; more formally,
  $${\left(A⊗B\right)}_{ij}=\left\{ \begin{array}{ll} 1\text{ iff} & A_{ij}=B_{ij}=1\\ 0 & \text{otherwise}. \end{array}\right.$$

At the widefield level, we focused on the overlap between the inflammatory zones as defined by our algorithm and by the manual operator. We, therefore, defined

— false positives *M*_P_: the fraction of pixels found as inflammatory by the algorithm, but not by the operator, formally $M_{P}=(\sum_{i,j}(1-T)⊗I)/S$,
— false negatives *M*_N_: the fraction of pixels found not to be inflammatory by the algorithm, but classified as inflammatory by the operator, $M_{N}=(\sum_{i,j}T⊗(1-I))\text{/}S$,
— mislabelled pixels: the fraction of pixels labelled differently by the algorithm and the manual operator, equivalent to the sum of false positives and false negatives, $M=(\sum_{i,j}[T⊗(1-I))+(1-T)⊗I])\text{/}S=M_{P}+M_{N}$.

In addition, we were interested in correctly identifying individual zones of inflammation. We defined a *zone* as a continuous, connected region of white pixels in an image (figure 2*d*), potentially representative of an inflammatory focus when found in *I* and certainly when found in *T*. We wished to measure whether the zones found by the algorithm in *I* could be attributed to a zone defined by the manual operator, *T*. Conversely, we also wished to measure the extent to which any true inflammatory zones as defined in *T* were not found by the algorithm. Let *Z*[*A*] denote the set of all inflammatory zones in image *A*. If *z* is a zone in image *A*, that is, *z* ∈ *Z*[*A*], then let *A_z_* denote a black image where only those pixels belonging to *z* are white, such that *A_z_* represents an image where only zone *z* is present. Then, we introduce

— *F*_A_, the fraction of inflammatory zones found by the algorithm which share at least 80% of their pixels with inflammatory zones described by the operator, formally
  $$F_{A}=\frac{\sum_{z\in Z[I]}1\left[\sum_{i,j}(I_{z}⊗T)\text{/}\sum_{i,j}1[I_{z}=1]\right]}{\left||Z[I]|\right|},$$
  where 1[·] denotes the indicator function and ||·|| denotes cardinality, or the number of elements in the set,
— *F*_G_, the fraction of inflammatory zones defined by the operator which are at least 50% present in the algorithm-generated binary images, formally
  $$F_{G}=\frac{\sum_{z\in Z[T]}1\left[\sum_{i,j}(I⊗T_{z})/\sum_{i,j}1[T_{z}=1]\right]}{\left||Z[T]|\right|}.$$

The thresholds (80% for *F*_A_ and 50% for *F*_G_) are chosen arbitrarily. In the case of *F*_A_, we set a rather high threshold to validate the method while allowing some variability as the human operator may have difficulty precisely defining the borders of an inflammatory focus. The value is set lower for *F*_G_ because inflammatory foci generally include veins, potentially large and empty, which may be selected by the human operator and discarded by the algorithm. A higher threshold may thus artificially lower the score even if the algorithm performs well. It is possible to think of *F*_A_ as a validity score and *F*_G_, its complementary measure, as a recall score.

At the narrowfield level, validation was simpler. We compared the surface area of the central inflammatory zone found by the algorithm directly with that found by the manual operator. We also computed the nuclear density, both inside the region used by the operator, and also across the whole image, excluding the central inflammatory zone. The former allows us to check agreement between the operator and our algorithm; the latter verifies whether sampling a subset of the image to count nuclei, as is done in pathology, may lead to error in estimating overall cell counts or densities.

All of the image processing in the automated algorithms was developed in Python, primarily using the ‘scikit-image' package [35].

## 5. Results

**Figure 4.**
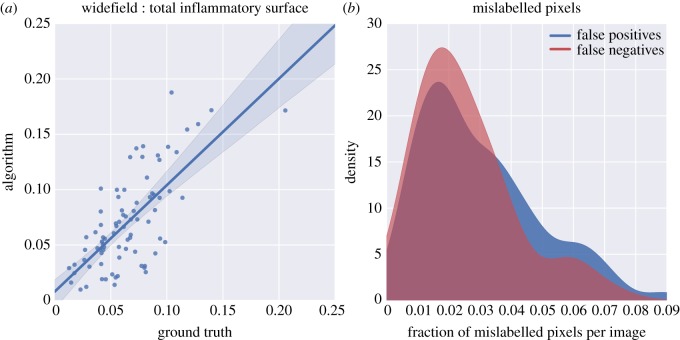
Comparison of inflammatory foci identified by the image processing algorithm versus an expert human operator. (*a*) Total fraction of inflammatory pixels per widefield image, with the ground truth shown against automated results. *R*^2^ = 0.56, *p *= 3.1 × 10^−18^. The slope of the robust regression line is near unity, at 0.96; bootstrapped confidence intervals are shown at the 95% level. This panel uses equal axes with grid squares of equal values, and the diagonal of the figure represents the 1 : 1 line. (*b*) Distributions of the fractions of false-positive and false-negative rates across images.

The image processing algorithm applied to images at the widefield level performed very well compared to a trained human operator. For example, in terms of total inflammatory surface per image, a good fit is found between the measure of total inflammatory surface by the algorithm and the manual operator, with a coefficient of determination of *R*^2^ = 0.56 and a slope of 0.96 (figure 4*a*) from a robust linear regression (M-estimation using Huber's T function). We also found low average false-positive and false-negative rates, at 3.1% and 2.5%, respectively, for a total of only 5.6% mislabelled pixels averaged over all images (table 1 and figure 4*b*).

**Table 1.** Widefield algorithm score statistics.

statistic	algorithm score	ideal value
*M*_P_	0.031	0
*M*_N_	0.025	0
*M*	0.056	0
*F*_A_	0.979	1
*F*_G_	0.744	1

Of the individual inflammatory zones found by our algorithm in widefield images, 98% (724/739) have at least 80% of their surface area in common with operator-defined inflammatory zones; this indicates that nearly all inflammatory zones found by our algorithm are, indeed, inflammatory regions, as defined by the operator. The algorithm discovers 74% (582/787) of operator-defined inflammatory zones, as per our 50% coverage criterion.

**Figure 5.**
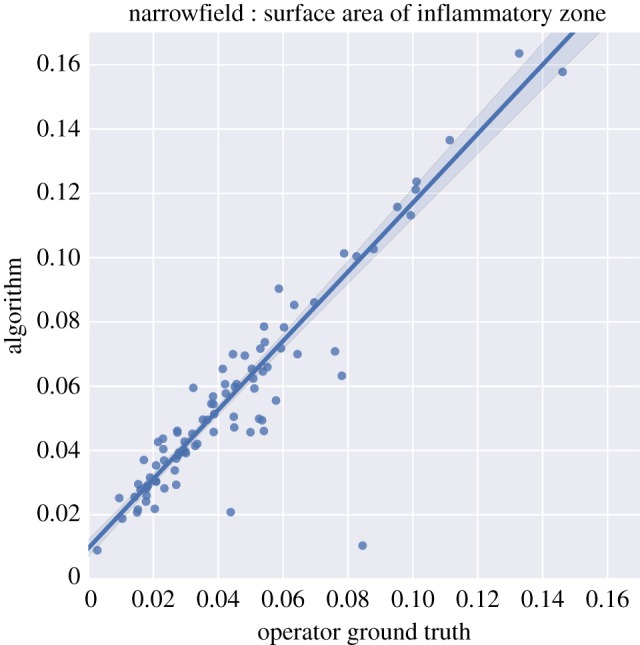
Comparison of the area of inflammatory foci estimated by the image processing algorithm versus an expert human operator. The surface area of the primary inflammatory zone as a proportion of the total surface in the narrowfield image. The area found by the algorithm is plotted against the ground truth, as defined by the human operator. *R*^2^ = 0.83, *p *= 2.5 × 10^−34^. The slope of the regression line is 1.07; bootstrapped confidence intervals are shown at the 95% level. The figure uses equal axes with grid squares of equal values, and the diagonal of the figure represents the 1 : 1 line.

At the narrowfield, the agreement between the human operator and the automated image processing algorithm for the surface area of inflammatory zones proved excellent (figure 5). Apart from a small number of outliers—which may, again, have been caused by the algorithm not labelling veins as inflammatory zones—the algorithm performs extremely well, with nearly a one-to-one mapping in terms of slope (slope = 1.07), and a coefficient of determination *R*^2^ of 0.88.

**Figure 6.**
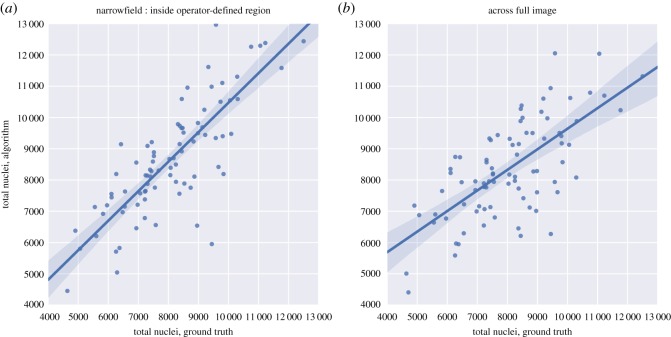
Comparison of nuclear density estimates for subsampled versus entire images. Nuclei per unit area in narrowfield images. (*a*) The density computed inside the operator-selected region. *R*^2^ = 0.65, *p *= 1.1 × 10^−16^. The slope of the regression line is 0.93; bootstrapped confidence intervals are shown at the 95% level. (*b*) Density computed for the entire image *R*^2^ = 0.47, *p *= 3.5 × 10^−13^. The slope of the regression line is 0.66; bootstrapped confidence intervals are shown at the 95% level. Both panels use equal axes with grid squares of equal values, and the diagonal of the figure represents the 1 : 1 line.

Finally, we calculated nuclear density, both inside the operator-selected region and across the entirety of each image (including the operator-selected region), and compared these quantities with the densities as defined by the operator inside the subset of the image. Densities of nuclei reported by the algorithm showed a relatively good fit compared with densities counted inside of the operator-selected subset of the image, with a coefficient of determination *R*^2^ of 0.65 and the slope of the regression line at 0.93 (figure 6*a*). By contrast, *R*^2^ dropped to 0.47 when we computed nuclear densities across the full image, with the slope of the fit equal to 0.66 (figure 6*b*). This may indicate that the operator may be biased in the selection of a ‘representative' area and that background nuclei density (i.e. outside the portal, potentially inflammatory, area) is not homogeneous.

Generating the ground truth data took an operator over five and a half hours of repetitive tasks, which may cause errors in accuracy and reproducibility, and limits the size of the datasets that can be generated in reasonable time [36,37]. By contrast, automated image processing methods are entirely reproducible, and can be much faster: fully processing the 81 widefield and 88 narrowfield images in our dataset to quantify nuclear density per inflammatory zone and total inflammatory surface area per image took only 32 min on a laptop computer. With the same algorithms being applied to a number of images, this type of processing is highly parallelizable: one image could be processed per core, such that hundreds of images, if not more, could be processed simultaneously on a supercomputer to drastically reduce computation times.

## 6. Discussion

Gross pathology of the liver provides essential information to determine the aetiology of pathologic processes contributing to mortality in wild animals experiencing infectious and nutritional challenges [38], including the Soay sheep population studied here [22]. The degree and distribution of hepatic inflammatory foci are particularly important variables in the evaluation of the overall health of the tissue and can only be accurately determined at the cellular level. Here, we report the use of tailored image processing algorithms, combining a number of well-established techniques, to quantify these aspects of inflammation in histological liver sections, rendered particularly variable by the constraints of fieldwork in remote places.

In general, we found strong correlations between the statistics of the operator-evaluated images and those automatically generated by our image processing algorithms. At the widefield level, low false-positive and false-negative rates indicate that inflammatory zones can be robustly found using a single algorithm applied to a high-variance dataset. Total inflammatory surface area per image matched reasonably well, with a coefficient of determination of *R*^2^ = 0.55. This is perhaps indicative of the breadth of variability in the dataset: for an error rate of 4.9% per pixel, a higher value for *R*^2^ would be expected if the errors were independent across images. This implies that, for a given image, the image processing algorithm consistently underestimates or overestimates the presence of inflammatory zones in a given image. Inconsistent uptake of stains or variations in the thickness of tissue sections may result in this sort of consistent bias. Nonetheless, nearly all inflammatory zones (98%) found by the algorithm are confirmed by the operator-based evaluation, demonstrating the high quality of the information computed by our automated workflow. The lower score of 74% for the fraction of inflammatory zones that are detected by both the human operator and the algorithm should be interpreted in light of its definition: we counted inflammatory zones as detected only if the algorithm found more than half of their surface area. One potential discrepancy between the operator definition and the algorithm definition of inflammatory foci is whether they included potentially large portal veins (see figure 1, for examples). Because the algorithm does not include veins, we can expect some cases where our overlap criterion would not be met because of the size of the vein. Indeed, should we reduce the requirement for detection to one-quarter of the surface area, algorithm–operator agreement increases to 86%.

At the narrowfield level, the image processing algorithm detected the surface area of an inflammatory focus more accurately (*R*^2^ = 0.88 with the manual operator). Of the 88 images, only three points were outliers, all underestimates of inflammatory surface area on the part of the algorithm. Similar to the widefield, these few underestimations could be attributed to the presence of large, empty veins not being included in the area by the algorithm but being selected by the human operator as integral parts of the inflammatory focus. The better correlation at the narrowfield compared to the widefield could be attributed to the use of different contour-detection methods: the entropy-based technique used at the narrowfield is indeed more reliable than the colour segmentation used at the widefield, thus explaining the better correlation with the operator at the narrowfield.

We also found strong algorithm–operator agreement for the density of nuclei inside the operator-defined region of the image, indicating that automated image processing methods can extract counts of nuclei effectively from images, despite considerable variation in nuclear staining and tissue appearance. However, for nuclear densities computed across the full image (excluding the central inflammatory zone), there is more discrepancy between the human evaluation and the algorithm's output. This could be suggestive of non-uniform nuclear densities across the image. The zone selected by the operator for counting nuclei could be, in fact, not entirely representative of nuclear density everywhere in the background tissue on the image. Cell density is non-trivial to estimate visually, and the operator may select a region where density looks approximately representative, but also where nuclei are well-defined and clearly separated. Regions with high density outside this region, where nuclei partially overlap, may cause an underestimation bias from the algorithm. However, operator bias in selecting the area in which to count nuclei may be considerable. Areas where nuclei are clearly defined, distinct and well stained may be more attractive to the operator, and thus more likely to be selected. Furthermore, while the algorithms may not segment overlapping nuclei perfectly, this effect is consistent, whereas operator interpretation is fundamentally subjective, suffering from bias from a variety of sources [39]. Even when scored by the same operator, significant variation and bias can be found between analyses (electronic supplementary material, figures S2 and S3). Overall, this indicates that algorithm-based detection of nuclei is more repeatable, and suggests it allows for larger, potentially more representative, areas to be scanned than are standardly measured by a human pathologist. Whether these improved counts may improve our ability to predict the extent of liver dysfunction or patient prognosis remains an open question. Applying these techniques in cases of progressive liver diseases would thus be of interest.

In terms of speed, the automatic approach to extracting data from image datasets is vastly faster than manual image analysis. We report an order of magnitude difference in the time required to analyse structural information in images; this was run on a single core of a standard laptop computer. The highly independent nature of image processing means that such analysis can easily be distributed on supercomputing clusters, with speed-ups linear in the number of cores used to process the images. Our algorithms took, on average, 5 s to process each image at the widefield level, and less than one minute at the narrowfield level. This approach also eliminates operator bias or fatigue; significant inter-operator bias can be found in traditional histological scoring, as a function of a number of variables that are not present in automated image processing [3].

Refinements can be made to the image processing algorithms to reduce the error in measures made during information extraction. Of considerable importance to this task is high-quality validation information. Our algorithms were validated against an operator-defined ground truth; however, evidence points towards scoring variation between operators [39], and thus, having a number of operators perform the validation tasks would be invaluable to both parameterizing the algorithms and in determining how much error is truly being made by the information extraction. Given a dataset of a sufficient size, parameters could even be inferred against a training set and validated on the remaining images.

In conclusion, we have demonstrated that high-quality, quantitative structural information can be extracted from noisy datasets, rapidly and robustly. Complete automation offers reproducibility and speed, generating fully quantitative information from image data. These measures are amenable to immediate statistical analysis and can relate to aspects of the image that manual assessment may not be able to capture, thus overcoming operator limitations. The algorithm may thus perform better than, and certainly performs faster than, a human pathologist. Our results show that a number of structural features can be computed accurately, without the need for any operator input. These methods have potential in any experimental or clinical setting where structural, spatial or visual information is of relevance. While our study focused on wild sheep liver subjected to malnutrition, similar algorithms may be readily implemented for other species, organs and pathologies. In many clinical diagnostic settings and in certain experimental studies, such methods are already being developed to automate data analysis. As we have shown, however, such methods are not limited to settings where the data are optimal in terms of quality: even in high-variation datasets, such as the ones obtained for wild species in ecological settings, image processing methods can be developed for robustness.

## Supplementary Material

Supplemental figures

## Data accessibility

The image dataset and the algorithm and operator numerical data are available from the Dryad Digital Repository (http://dx.doi.org/10.5061/dryad.pn6jr) [40]. The code is freely available on Github at http://github.com/qcaudron/quantitative_histology. The file ‘process_images.py' contains the Python code, and this code is published under the MIT licence.
