# Supplementary material for: Robust extraction of quantitative structural information from high-variance histological images of livers from necropsied Soay sheep
Source: R Soc Open Sci. 2017 Jul 19;4(7):170111. doi: 10.1098/rsos.170111 (PMC5541533; doi:10.1098/rsos.170111)
Supplement: Supplemental figures [file rsos170111supp1.docx]

**Supplementary Information**


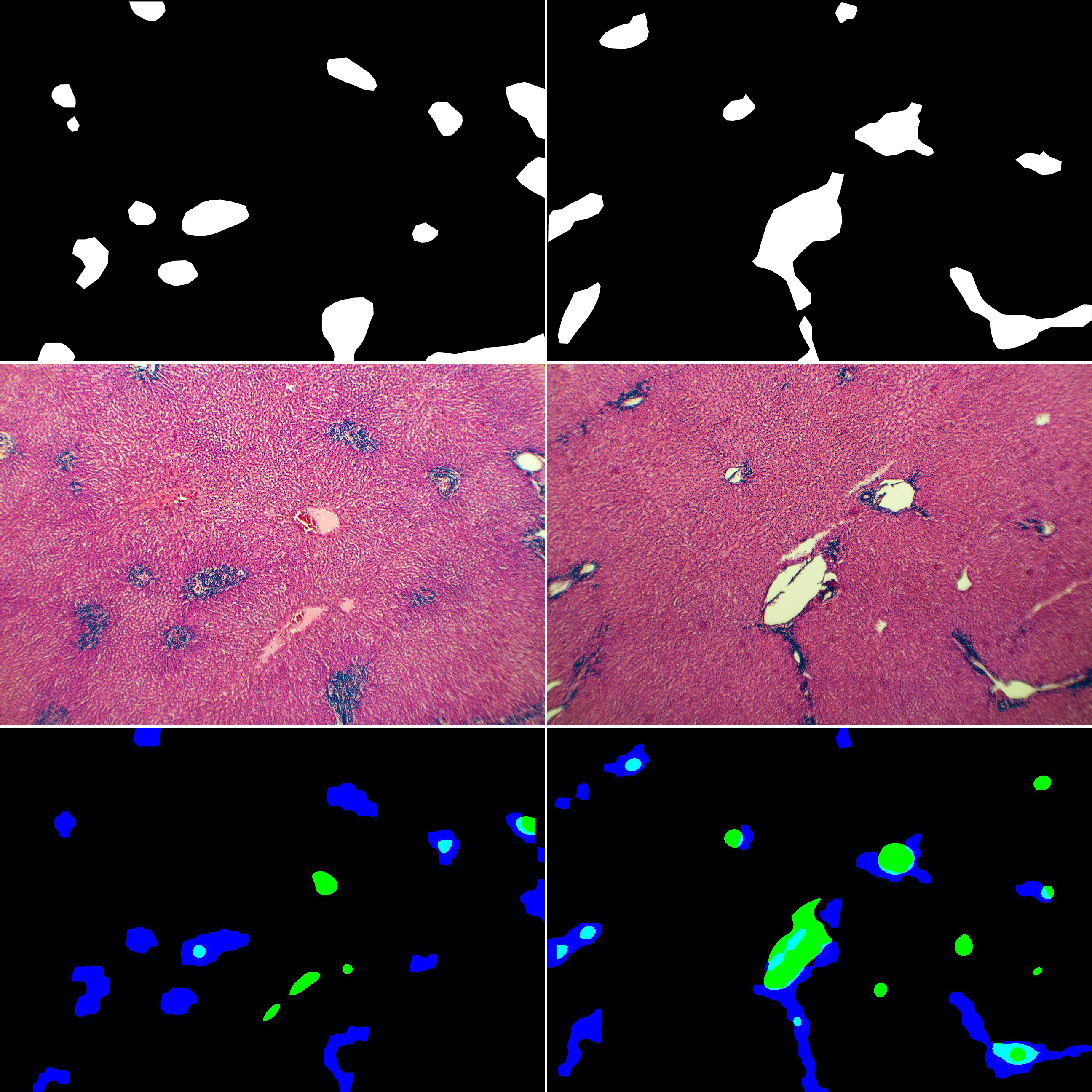


Figure S1. Top : Image *T*, the binary images generated by a human operator outlining zones of inflammation. Middle : the original images. Bottom : Images *I*, the inflammatory zones (blue) and veins (green) as found by the image processing algorithm.


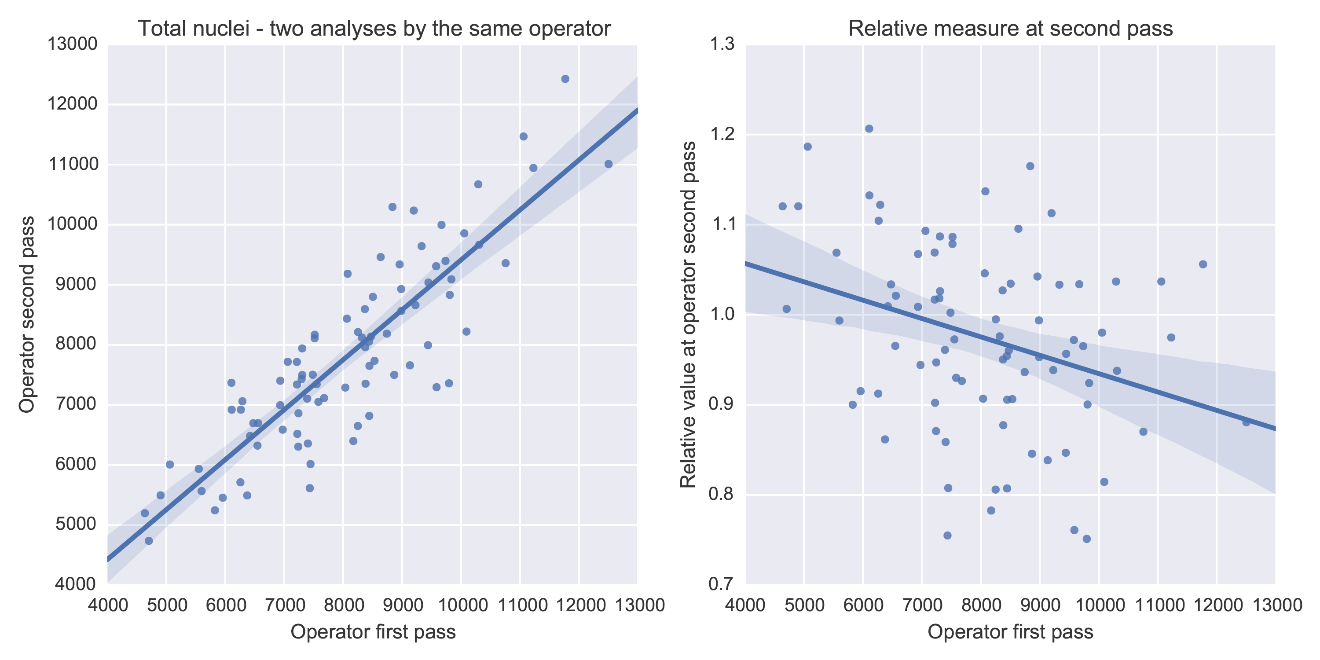


Figure S2. Left : comparison between two counts of total nuclei in the same set of narrowfield images, performed by the same operator; the slope is 0.83. $\boldsymbol{R}^{\boldsymbol{2}}\boldsymbol{=0.74, p=3.2\times}\boldsymbol{10}^{\boldsymbol{-26}}$. Right : the value of the measure during the operator’s second pass, relative to the value at the first pass; for perfect agreement between analyses, all points would have a relative value of one. A significant negative slope is found, indicating that the operator counted fewer nuclei in the second pass per nucleus found in the first pass. $\boldsymbol{R}^{\boldsymbol{2}}\boldsymbol{=0.10, p=2.9\times}\boldsymbol{10}^{\boldsymbol{-3}}$.


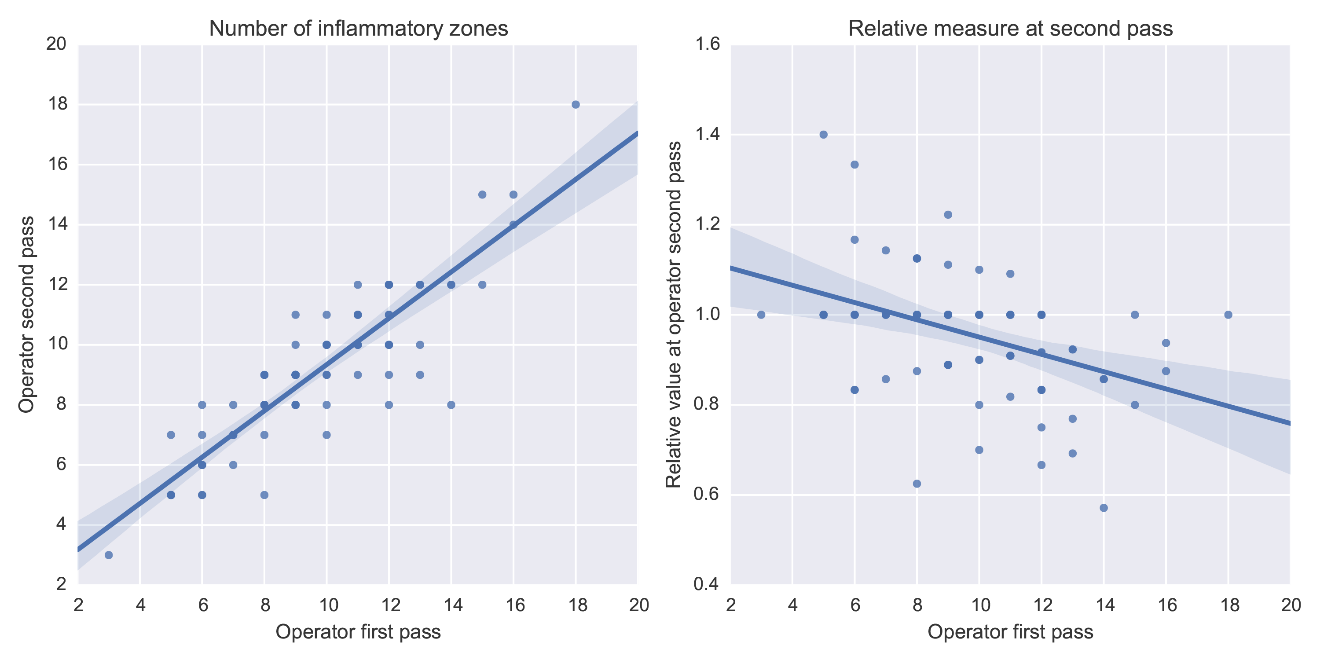


Figure S3. Left : comparison between two counts of inflammatory foci in the same set of widefield images, performed by the same operator, with a slope of 0.77. $\boldsymbol{R}^{\boldsymbol{2}}\boldsymbol{=0.78, p=1.1\times}\boldsymbol{10}^{\boldsymbol{-27}}$. Right : the value of the measure during the operator’s second pass, relative to the value at the first pass; for perfect agreement between analyses, all points would have a relative value of one. A significant negative slope is found, indicating that the operator counted fewer inflammatory foci in the second pass per focus found in the first pass. $\boldsymbol{R}^{\boldsymbol{2}}\boldsymbol{=0.17, p=1.2\times}\boldsymbol{10}^{\boldsymbol{-4}}$.

**Image processing parameters – widefield segmentation of inflammatory zones**

Image deconvolution

The color convolution matrix, as described by Landini (<http://www.mecourse.com/landinig/software/cdeconv/cdeconv.html> ), has the values

$$\begin{matrix} -0.15708943 & 1.24610358 & -0.36185705 \\ -1.356457 & -0.31820437 & 2.25069365 \\ 2.1527013 & -0.20198654 & -1.32520551 \end{matrix}$$

Sigmoid transform

The following transform was used to increase contrast in the image :

$$O=\frac{1}{1+e^{10(0.6-I)}}$$

where *O* is the output image, and *I* is the input image, with pixel values between 0 and 1.

Adaptive threshold

An adaptive threshold binarises each pixel according to whether its value is greater or less than a local threshold. That local threshold is the mean of all pixel values in a 75-by-75 pixel square around the pixel. At this stage in the processing, we apply an offset to the threshold of -0.12.

**Image processing parameters – narrowfield segmentation of nuclei**

Sigmoid transform

The following transform was used to increase contrast in the image :

$$O=\frac{1}{1+e^{20(0.6-I)}}$$

where *O* is the output image, and *I* is the input image, with pixel values between 0 and 1.

Adaptive threshold

Pixels that were brighter than their local threshold ( the mean of all pixel values in a 151-by-151 pixel square around the pixel ) became white; otherwise, they became black.

**Image processing parameters – narrowfield detection of inflammatory focus contour**

Entropy

Local entropy was found starting with an image blurred by a Gaussian filter with a standard deviation of 61 pixels; we denote this image *B*. Entropy was computed over a local 3-pixel-radius disk, and the result was again blurred with a Gaussian filter, this time with a standard deviation of 75 pixels. The result is denoted *E*.

Entropy was nonlinearly combined with the starting blurred image according to

$$O=E( 1+B)$$

where *O* is the output image where areas of high luminosity correlate with areas of nuclear density.

Contours

From *O* as defined immediately above, contours were defined as the central-most constant-valued contour where luminosity was at a value of 0.5.
